# Be Courteous

**Published:** 1855-11

**Authors:** 


### BE COURTEOUS.

Does a lady ever ride in an omnibus or a city rail-car? Women do often—and now and then a lady may, when impelled by some emergency of rain, or mud, or cash. The manner in which women take the seats vacated by gentlemen, who have in consequence to stand the remainder of the trip, is anything but confirmatory of the fact that our fair countrywomen, as a class, know what common courtesy is, practically. In a daily car-riding of five or six years, we cannot remember as many instances of a ladylike acceptance of a proffered seat. It is almost universal, that a gentleman's place is taken without the slightest acknowledgment by word, or look, or gesture, that a benefit has been conferred and received, and yet it is a very great accommodation; for to stand in the passage-way, while the cars are in motion for a dozen squares or so, the centre of thirty pairs of eyes, is very short of purgatorial; and being such an accommodation, the smallest kind of a remuneration would be a word, or look, or gesture of felt indebtedness. The perseverance which New York gentlemen exhibit, in instantaneously quitting their seats when a car is crowded, and a woman enters, is highly creditable to their manliness and chivalry.

We suggest, as a remedy, that all the "boarding-schools," "day-schools," and "institutes," which have the prefix *Female,* hold a convention immediately, if not sooner, for the purpose of debating the question, whether or not a Professor of "Politeness" might not be appointed to universal advantage, whose duty it should be to "give lessons in politeness" to every young girl in the school, from her entrance until her exit from the establishment. We have seen tottering gray-headed men resign their seats to young women, and not a smile, or curtsey, or "thank you," ever escape from their lips. Shame on the superficial, inadequate, corrupting and debasing system of "female boarding-schools" and "institutes" as a class, whose absorbing object is not to prepare the girls committed to their care to become helping wives, intelligent mothers, discreet matrons of a household, and ornaments in useful and benevolent society, but to make money, and return therefor a painted flower, a gilded time-piece, with no enduring quality but the brass of which it is chiefly composed. How sigh we for the wives, the mothers, the daughters of a by-gone age!

There is a name, now passed away, we love to think upon! a synonym, a representative in his age, of all that was honorable in his dealing, courteous in his deportment, manly in his bearing, and Christian in his heart,--a fine Virginia gentleman of the old school was *James Harper*. He once related to us the following incident.

"Some years ago, an old woman entered a public conveyance in Broadway: it was raining, and there was no vacant seat. I instantly offered her mine; she declined, and in a manner which showed that she felt she had no claim for the seat, nor to such an evidence of consideration from a stranger. I insisted, and, as if fearing to wound my feelings by a further refusal, she took it, with a courteous expression of her obligation. When she wanted to leave the conveyance, it stopped in a muddy part of the street, and feeling assured that I was with a lady, I did not hesitate to pass out before her, and hand her to the side-walk. I then returned to my seat doubly gratified: first, in having it in my power to oblige a lady; and, second, in seeing that it was appreciated—not a common thing, doctor, now-a-days:" as he turned away with one of his hearty, full-souled laughs.

But who was the lady?

"I learned afterwards, that it was MRS. ALEXANDER HAMILTON."

---

KIND WORDS—are among the brightest flowers of earth; they convert the humblest home into a paradise; therefore use them, especially around the fire-side circle; they are jewels beyond price, they heal the wounded heart and make the weighed-down spirit glad. ANON.

